# Information Transmission in Cercal Giant Interneurons Is Unaffected by Axonal Conduction Noise

**DOI:** 10.1371/journal.pone.0030115

**Published:** 2012-01-12

**Authors:** Zane N. Aldworth, John A. Bender, John P. Miller

**Affiliations:** Center for Computational Biology, Montana State University, Bozeman, Montana, United States of America; Université de Montréal, Canada

## Abstract

What are the fundamental constraints on the precision and accuracy with which nervous systems can process information? One constraint must reflect the intrinsic “noisiness” of the mechanisms that transmit information between nerve cells. Most neurons transmit information through the probabilistic generation and propagation of spikes along axons, and recent modeling studies suggest that noise from spike propagation might pose a significant constraint on the rate at which information could be transmitted between neurons. However, the magnitude and functional significance of this noise source in actual cells remains poorly understood. We measured variability in conduction time along the axons of identified neurons in the cercal sensory system of the cricket *Acheta domesticus*, and used information theory to calculate the effects of this variability on sensory coding. We found that the variability in spike propagation speed is not large enough to constrain the accuracy of neural encoding in this system.

## Introduction

An important problem in neuroscience concerns sensory coding: how nervous systems represent information about the sensory environment. What determines the fidelity, specificity, and reliability with which sensory coding can be accomplished? When examined more generally, the question becomes: “How much information about the sensory environment is represented within nervous systems?”

One way that this problem has been approached has been through the use of information theory. As originally formulated by Shannon, this approach examines the amount of information that can be transmitted along a noisy channel [1]. This approach has been successfully applied to questions in neuroscience for nearly 60 years [2]–[4].

Most studies employing information theoretic analysis in neuroscience have relied on measurements of variability in the spike train at a single location, usually near the spike initiation zone (SIZ). Implicit in this approach is the assumption that all noise sources between encoder and decoder act prior to the conduction of spikes along the axon. However, numerous studies dating back to the earliest neurophysiological experiments have established that the axon itself does not act as a deterministic channel, but is instead subject to several sources of noise, all of which affect the patterning of spikes [5]–[18]. This noise accumulates downstream of the SIZ, prior to contact with postsynaptic neurons. Recent modeling work has suggested that up to 27% of the information encoded at the SIZ can be lost during conduction along the axon [19].

To determine the effects of conduction noise on information transmission we examined projecting interneurons of the cercal sensory system of the house cricket, *Acheta domesticus* [20]–[23]. We combined intracellular and extracellular recording techniques to monitor the neurons' spontaneous activity as well as their response to sensory stimulation. We characterized several sources of noise that arise during action potential (AP) conduction under these conditions, including temporal uncertainty arising from AP conduction (transmission jitter), AP acceleration due to a supernormal period, AP deceleration due to refractory effects, and AP conduction failures. We found that the accumulation of noise effects, though measurable, did not significantly change the quality of information transmission in this system. Further, modeling of the information loss as a function of these noise sources revealed that information transmission remains largely unaffected over a wide parameter range.

## Materials and Methods

### Preparation, Electrophysiology, and Stimulation

All experimental animals were of the species *Acheta domesticus* obtained from a commercial supplier (Basset's Cricket Ranch, Visalia California). Animals were fed cat food and water in the laboratory, and maintained on a 12 hour light-dark cycle at 21°C. Experiments were conducted on 8 female crickets that had undergone their final molt within the previous 24 hours. Animals were anaesthetized by placing them on ice for 5-10 minutes, after which their legs, ovipositor, wings, gut, reproductive organs, and fatty tissue were removed. The preparation was pinned to a disk of silicone elastomer, and all incisions were sealed with petroleum jelly. The abdominal cavity was perfused with hypotonic cricket saline [24], and a small steel platform was inserted under the terminal abdominal ganglion (TAG) for stability. For dual intracellular recordings (n = 4), a second steel platform was inserted under the connectives between the metathoracic (T3) ganglion and the first unattached abdominal ganglion (A3).

Intracellular recordings were made from neurons 10-2a (n = 4) and 10-3a (n = 4), two bilaterally symmetric pairs of giant projecting interneurons with cell bodies in the TAG [22]. These neurons have axons of approximately 10-20 µm diameter extending the length of the ventral nerve cord to the brain [21]–[23], [25], with extensive axonal arborizations in the thoracic ganglia [23], [25].

Sharp intracellular electrode penetration into the axons of these neurons was facilitated by first applying protease solution (Sigma-Aldrich, P5147, St Louis, MO). Axons were impaled at the point where they exited the TAG in the ventral nerve cord, as well as at the connective between T3 and A3 for the dual intracellular experiments. Electrodes were filled with a mixture of 2% Neurobiotin (Vector Laboratories, SP1120) and 3M KCl, yielding electrode resistances between 2 and 10 MΩ. During neural recordings the Neurobiotin passively entered the neuron, and following the experiment the neurobiotin in the neuron was conjugated using an ABC-DAB reaction (DAKOCytomation K0377, and Vector Laboratories SK4100, respectively) for morphological identification. Two of the eight neurons were identified morphologically, while in the other six neurons identification was based on physiological properties. For intracellular-extracellular experiments, a chlorided silver wire was fashioned into a hook electrode, and sealed around the connective between T3 and A3 using a petroleum jelly-mineral oil mixture.

Experiments were performed in a previously described stimulation system [26], in which air particle displacement generated by stereo speakers stimulated the filiform hairs on the crickets' cerci. Each filiform hair is innervated by an afferent neuron that makes direct excitatory synaptic contact with the giant projecting interneurons. All stimuli consisted of half-cosine air puffs for generating tuning curves for the dual intracellular experiments, and 20 repeated presentations of a 10-second, 5–300 Hz band passed white noise stimuli for the intracellular-extracellular experiments. Intracellular membrane voltages were sent to an intracellular amplifier (npi SEC-05L, Tamm, Germany), while extracellular voltages were sent to a Data model 2124 differential amplifier (Fort Collins, CO), using gain settings of 300-3000x and band pass filtering from 200 to 10000 Hz. During experiments the physiological and stimulus voltages were sampled at either 10 (n = 2) or 200 (n = 6) kHz and recorded on a Windows XP computer running proprietary LabVIEW software.

### Quantification of Conduction Noise

Four different noise sources with respect to the velocity of action potential conduction velocity were characterized. These were the jitter around mean conduction time for action potentials to reach the T3 ganglion, expansive and compressive non-linearities whereby the second action potential of a doublet traveled along the axon at either a slower or faster velocity, respectively, relative to the first action potential, and the probability that an action potential would fail during conduction along the axon (conduction failure).

Transmission jitter was assessed in two different ways, depending on whether the recording used two intracellular electrodes or an intracellular/extracellular electrode combination. For the dual intracellular recordings, the timing of each action potential at each recording site was determined by finding the peak in the intracellular waveform above a user-defined threshold. The 1 ms waveform surrounding the estimated peak was fit with a fourth-order polynomial, and the peak of this polynomial function was used as the sub-sampling precision timing of the action potential. For recordings in which there was a linear increase in transmission time throughout the experiment, the linear trend was removed using the detrend function in Matlab. The transmission jitter was estimated as the standard deviation in conduction time between the two electrodes. Note that in order to account for the observed expansive non-linearity (see below), the exponential decay function relating change in conduction time to preceding inter-spike-interval (ISI) was subtracted from the arrival time of the second spike before estimating jitter.

**Figure 1:**
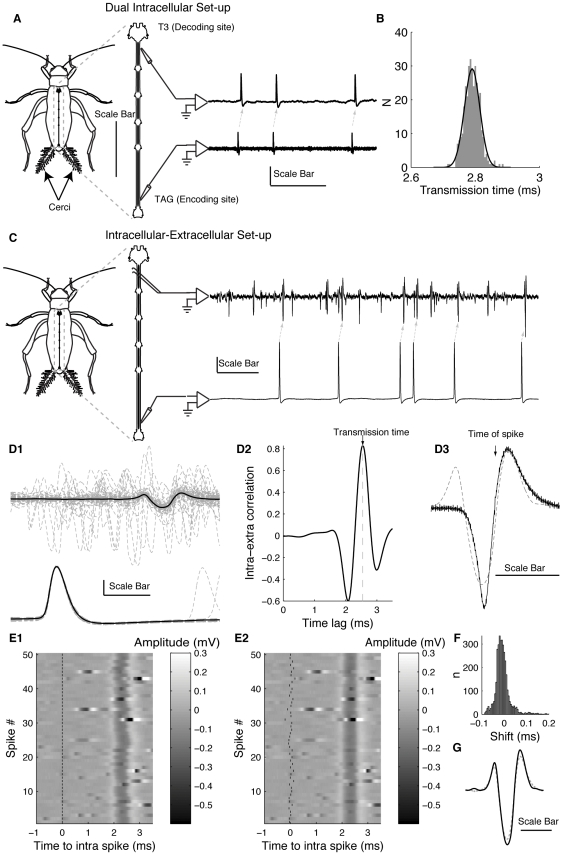
Experimental set-up and measurement of transmission jitter. ***A***, Outline of cricket, cerci and filiform hairs, and ventral nerve cord, with approximate placing of recording electrodes as well as simultaneous intracellular recording from encoding (lower trace) and decoding (upper trace) sites in a recorded neuron. Scale bars: 1 cm (full animal), 3 mm (nerve cord); phys. recording- 50 ms, 2 mV (lower trace) and 10 mV (upper trace). *B*, Histogram showing distribution of transmission time of spikes from recording shown in *A*, as well as Gaussian fit (dark line). *C*, Intracellular-extracellular experimental set-up, convention as in *A*. Scale bars: horizontal, 25 ms; vertical, upper trace, 100 µV; vertical, lower trace, 20 mV. *D1*, ∼100 samples of intracellular spikes (lower trace, dashed light grey lines) and corresponding extracellular waveforms (upper trace, dashed light grey lines). Mean waveforms are shown with solid black lines. Scale bars: horizontal, 1 ms; vertical, upper trace, 250 µV; vertical lower trace, 20 mV. *D2*, Cross-correlation between the negative temporal derivative of the mean intracellular spike waveform, and the mean extracellular waveform. Correlation is normalized so that autocorrelations at zero lag have values of 1. *D3*, Negative temporal derivative of mean intracellular waveform (black line) and extracellular waveform (dashed grey line), aligned by maximum lag indicated in *D2*. Time of spike (peak in intracellular waveform) is indicated with an arrow. Scale bar: 1 ms. *E1*, Raster plot of a selection of 50 extracellular waveforms, aligned according to time of intracellular spike (time of intracellular spike indicated by vertical lines at t = 0), with amplitude of extracellular spike indicated in greyscale. *E2*, Same raster shown in *E1*, with extracellular waveforms aligned through the dejittering algorithm (see Methods). *F*, Histogram of shift times for all 3556 spikes. Asymmetric distribution towards long positive shift times indicates non-linear transmission time for second spike of short ISIs. Standard deviation of shift times after correcting for nonlinearity (transmission jitter) was 24 µs. *G*, Change in mean extracellular waveform, before (dashed grey line) and after (solid black line) dejittering. Scale bar: 1 ms.

A necessarily different method was required for finding the jitter for the intracellular-extracellular recordings, depicted graphically in Figure 1D–1G. As a first step, the timing of the intracellular action potential at the TAG was found as for the dual intracellular case described above. The mean conduction velocity was then found by comparing the negative derivative of the mean intracellular waveform with a 5-ms window of the corresponding extracellular voltage, starting at the time of the intracellular action potential. The xcorr function in Matlab (Mathworks) was used to find the timing offset where the two mean waveforms coincided (Figure 1D). The jitter around this mean waveform was estimated through modification of the ‘dejittering’ algorithm which we previously developed to examine spike-triggered stimuli [27]. Briefly, the ensemble of extracellular waveforms surrounding the mean conduction time was extracted, and then each individual waveform was shifted in time by minimization of a Gaussian distance *d* according to
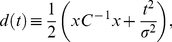
(1)where *x* is the residual between the specific extracellular waveform and the mean waveform across the ensemble, *C* is the covariance matrix of the ensemble, *σ* is the assumed variance of the jitter distribution (a parameter set at 0.15 ms- changing this parameter did not affect the results), and *t* is the specific shift time being tested. This distance *d* was calculated for the range of *t* values from -3*σ* to 3*σ*, and the value of *t* that minimized *d* was selected as the shift time for that action potential. The dejittering procedure and resultant jitter is depicted in Figure 1E–G. Two confounds occurred in this data that added difficulty to estimations of the propagation jitter. First, superposition of action potentials from other neurons recorded on the hook electrode sometimes affected the waveshape of individual extracellularly-recorded action potentials. Second, our dual intracellular data indicates that a small number of action potentials fail to conduct along the length of the axons. Both of these effects have the potential to lead to large outliers in the distribution of transmission jitters. To avoid these effects we removed as outliers all samples that had transmission time greater than four times the calculated sample jitter.

The expansive non-linearity was estimated by using the nlinfit function in Matlab to fit a standard exponential decay function to data expressing the conduction time of action potentials as a function of the preceding ISI. The exponential decay function was of the form
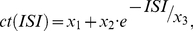
(2)where *ct(ISI)* was the conduction time as a function of the preceding ISI, and *x_1_* through *x_3_* are the fit parameters.

In cases where enough data was available, we estimated both the expansive and compressive non-linearity using a function of the form
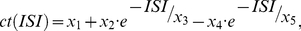
(3)where *x_4_* and *x_5_* are additional parameters for estimation of the compressive non-linearity.

The probability of conduction failure was only observable in the dual intracellular recordings, and was estimated by dividing the number of action potentials observed at the TAG that were not observed at T3 by the total number of action potentials observed at the TAG.

### Information-Theoretic Calculations

We adapted the model for calculating information rates on these neurons that we previously developed [28]. Briefly, we used exponential models for the onset jitter of doublet action potential patterns, and for the internal jitter of the doublets themselves to characterize the probability of spike patterns with specific ISIs at the TAG. The conditional entropy for the ISI, *H_C_*, was calculated according to
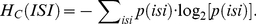
(4)


This yielded the conditional entropy per stimulus event. To transform this into a rate we weighted the conditional entropy by the probability of each ISI occurring (the normalized ISI histogram), and then multiplied this value by the firing rate of the cell. The unconditional or total response entropy rate was calculated using only the ISI histogram plugged into Eq. 4, multiplied by the firing rate. The mutual information of the models was estimated as the difference in the two entropy rates.

To determine the precision of the same patterns at the thoracic ganglia, we added a Gaussian noise source (mean = mean conduction time, standard deviation = observed transmission jitter) as well as the expansive non-linearity from Eq. 2. This produced new ISI probabilities representing spike patterns at the second recording site, from which information rates were estimated in the same way as for ISIs at the TAG, described above. Comparisons between information rates at the TAG and thoracic ganglia were used to assess the effect of conduction noise on information transmission in this system. We determined how the two noise sources could affect the transmission by calculating information rates using transmission jitter ranging from 0.001 to 1 ms, and using exponential decay terms (*x_2_* in Eq. 2) ranging from 0.001 to 1000 ms.

This model for information transmission was compared with the entropy and information rates calculated from our intracellular-extracellular data using the context tree weighting (CTW) method of Kennel and Shlens [29], [30] and the Spike Train Analysis Toolkit [31].

## Results

### Spike Conduction Velocity is Subject to Stochastic Jitter

Our experimental set-up was as follows: two electrodes were placed approximately 9 mm apart along an axon from an interneuron of cell class 10-2a (n = 4) or 10-3a (n = 4, Figure 1A). The neuron cell body and spike initiation zone for both cell classes are located in the TAG, near the first recording (intracellular) electrode. We refer to this as the *encoding site*, because this is where the collective activity of the presynaptic, afferent neurons is encoded into the action potentials that activate the interneuron. The first area of postsynaptic output for this interneuron is in the ganglion T3, near the second recording (intracellular or extracellular) electrode. We refer to this as the *decoding site*, because it is where the information carried in the spike train of the interneuron needs to be decoded by postsynaptic neurons. For experiments which included one intracellular and one extracellular electrode, a slightly different set-up was used, as depicted in Figure 1C. For the preparation depicted in Figure 1A, we recorded spontaneously evoked action potentials simultaneously at the encoding and decoding sites in order to assess variability during propagation.

Action potentials do not traverse the length of the axon at a single velocity, but instead show some variability in speed. This leads to a spread in the time it takes for a spike to travel from the encoding site to the decoding site. We refer to this spread in speed as transmission jitter, since it arises during conduction from encoding site to decoding site.

The distribution of transmission times for spontaneously-evoked action potentials was approximately Gaussian (Figure 1B, data shown with grey histogram, Gaussian fit shown with solid dark line). The standard deviation of this distribution (the transmission jitter) was 24 μs, with a mean conduction time of around 2.8 ms. This corresponds to about 9 μs of jitter per millisecond of conduction time, or a ∼1% error, compounding continuously. We measured this jitter in 8 neurons of class 10-2a and 10-3a, using both dual intracellular recordings (n = 4) and combined intracellular and extracellular recordings (n = 4, see methods). We found the transmission jitter to range from 19 to 74 μs over the ∼2.5 ms of transmission time, with a mean ± 1 SD of 32±19 µs (Table 1). This corresponds to a compounding percentage error of 1.35±0.81% (mean ± 1 SD).

**Table 1:** Transmission jitter and fit parameters for all 8 intra-intra and intra-extra experiments.

Cell ID	Cell type	Transmission jitter (µs)	Model parameters (Eqns. 2&3, main text)				
			*x_1_* (ms)	*x_2_* (ms)	*x_3_* (ms)	*x_4_* (ms)	*x_5_* (ms)
2006-05-14_1_a	10-2a	74	2.52	2.35	1.18		
2006-05-17_1_a	10-2a	42	1.90	1.72	1.48		
2006-05-19_1_a	10-3a	22	2.67	3.73	1.33		
2010-11-28_1_a	10-2a	25	1.82	1.62	0.98		
2004-08-18_1_a	10-3a	27	2.54	0.62	1.63	0.07	7.35
2004-08-26_1_b	10-3a	24	2.55	1.95	1.13	−0.34	1.13
2004-09-07_1_b	10-2a	19	2.46	0.95	1.48	0.09	5.74
071701_a	10-3a	19	2.90	1.82	1.56	0.05	6.51

### Spike Conduction Velocity is Subject to ISI-dependent Non-Linearities

In addition to the stochastic jitter described above, we also observed changes in conduction velocity that were dependent on the time since the previous spike. This was evident when we used sensory stimulation, which drove the spike rate up and led to more short duration ISIs. Specifically, for spikes that fall within the relative refractory period of a previous spike, some proportion of the voltage-gated sodium channels necessary for propagation remain in the inactive state, causing the second spike to decelerate relative to the speed of the first. This effect lengthens very short ISIs between their generation at the encoding site and their arrival at the synaptic arbors of the decoding site, while leaving longer ISIs unchanged. Although this effect has been reported previously [7], [9], [11]–[13], [32], [33], our study is the first to demonstrate it with action potentials generated by sensory stimulation rather than current injection. Since the distribution of ISIs is dependent on the stimulus (and the magnitude of the change in propagation velocity is in turn dependent on preceding ISI), our use of sensory stimulation allows us to draw stronger inferences about the relevance of this effect on neural coding in naturally behaving animals.

**Figure 2:**
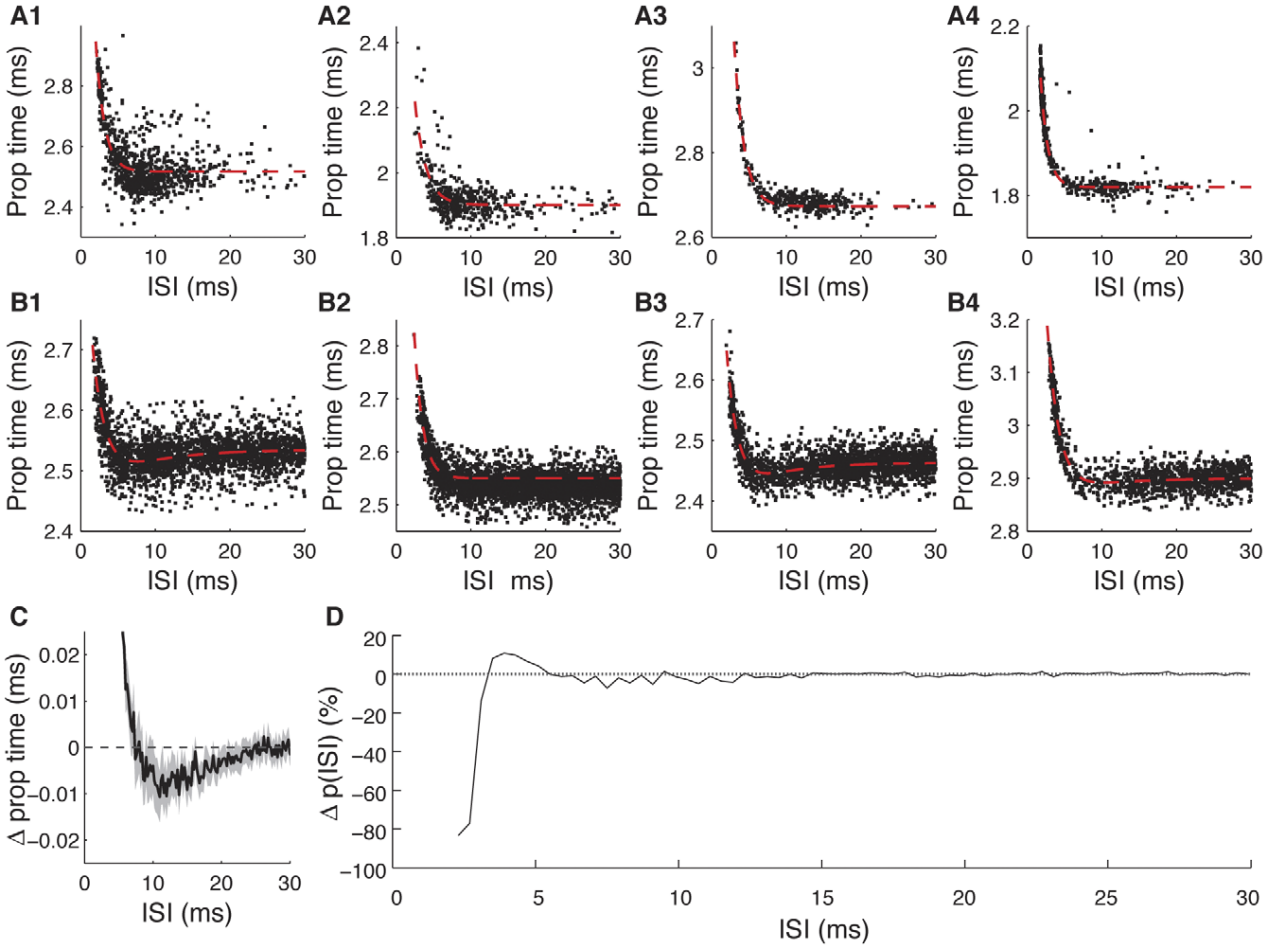
Expansive and compressive non-linearities in conduction velocity. *A*, The propagation time for all spikes from the four dual intracellular recordings, expressed as a function of the preceding ISI (black points). Also shown are the exponential fits to the data, using Eq. 2, (dashed red line, parameters in table 1). *B*, Data are presented as in *A*, but for four cells recorded with a combination of intracellular and extracellular electrodes. In these cases the fits are to a sum of exponentials function (Eq. 3, parameters in table 1). Note that in panel *B4* only every 10^th^ data point (of 157,775 recorded) is shown, for visual clarity. *C*, The mean change in propagation time as a function of ISI for the recording shown in *B4*, along with the 95% CI (grey envelope). Y axis is truncated at 0.25 ms. *D*, The percentage change in ISIs at the encoding vs. decoding recording electrodes, for the recording shown in *B4*.

The dependence of conduction velocity on the previous inter-spike interval (ISI) is shown in Figure 2. Figure 2A1–2A4 shows the conduction time of the second spike for all ISIs from four different dual-intracellular recordings, along with exponential fits to the data (dashed red lines, methods Eq. 2). In these cases, there appears to be an asymptotic conduction time for long ISIs (parameter *x_1_* from Eq. 2). However, for ISIs shorter than ∼7 ms, the second spikes required up to approximately 25% longer (∼0.5 ms out of 1.9 ms propagation time in A2) to propagate along the axon. We refer to this as an expansive non-linearity, since the ISI at the decoding end increases relative to the ISI at the encoding end. The parameter values for exponential fits for these four experiments are listed in the first four rows of Table 1.

Figure 2B1–2B4 shows results similar to those in Figure 2A1–2A4, but obtained in cells recorded with one intracellular and one extracellular electrode. The greater stability of the extracellular hook electrode allowed us to monitor cells for much longer, in the case of 2B4 for nearly 4.5 hours, sampling over 150,000 spikes from a single neuron. This large sample revealed not only variability caused by the deceleration of second spikes in short ISI pairs, but also an effective acceleration for second spikes of intermediate ISIs (∼10–20 ms). This acceleration is visible in Figure 2C, where the change from the steady-state propagation time is shown as a function of ISI for the neuron in panel 2B4 (envelope shows mean ± 95% CI). We refer to this acceleration as a compressive non-linearity, since the ISI at the decoding end decreases relative to the ISI at the encoding end.

Like the deceleration due to the relative refractory period, this effective acceleration of second spikes has also been observed before [8], [11], [14], [33], and is thought to be the result of an activity-dependent accumulation of potassium in the extracellular space around unmyelinated axons. We fit propagation time as a function of preceding ISI with a sum-of-exponentials (methods Eq. 3), in which the first exponential term (*x_2_* and *x_3_*) represented the effects of the deceleration for short ISIs and the second exponential term (*x_4_* and *x_5_*) represented the effects of acceleration for long ISIs. The parameter values for exponential fits for these four neurons are listed in rows 5–8 in Table 1.

Figure 2D shows how changes in conduction velocity affected the ISI distribution at the decoding site relative to the spike generation at the encoding site for the neuron in Figure 2B4, with the difference shown as a percentage. The net effect was to increase the probability of ISIs in the range from 3.6 to 5.2 ms (portion of curve>0), at the expense of decreasing the probability of shorter and longer ISIs.

### Spike Conduction Failure Rate is Low

**Figure 3:**
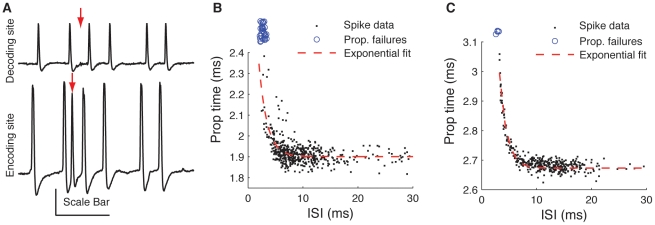
Conduction failures. *A*, Simultaneous intracellular recording from encoding (lower trace) and decoding (upper trace) sites in a single 10-2a neuron, showing an instance of an action potential which failed to propagate the length of the axon (red arrows). *B*, Distribution of spike propagation time as a function of ISI, along with exponential fit (dashed red line), as in figure 2. Also shown is the length of the preceding ISI for 32 action potentials that failed to propagate (blue circles, arbitrary ordinate position). *C*, Data are presented as in *B*, but for a different cell (class 10-3a). Scale bars: *A*, horizontal, 20 ms; vertical, 5 mV.

A third form of noise that we observed during action potential propagation was the failure of some spikes to travel the entire length of the neuron. Because of the difficulty in matching every spike in intracellular-extracellular recordings, this effect was only obvious in the dual intracellular experiments, and was evident when spikes on the encoding-site electrode were absent on the decoding-site electrode. An instance of such a conduction failure is shown in Figure 3A. Figure 3B shows the ISI that preceded all 32 conduction failures detected in the same recording as Figure 3A (failures denoted with blue circles, note that displayed height of failures along y axis is arbitrary), along with the conduction time of all 889 action potentials that did not fail. Figure 3C shows a similar plot for a second cell that had four failures out of 981 spikes observed at the encoding end. In both cells we note that failures only occur for the second spikes of very short ISIs. In all, only two out of our four dual intracellular recordings exhibited spike failures, accounting for 36 of the 4077 spikes (∼0.1%) observed at the encoding site across all four experiments.

### Information Transmission Rates are Unchanged Between Encoding and Decoding Ends

**Figure 4:**
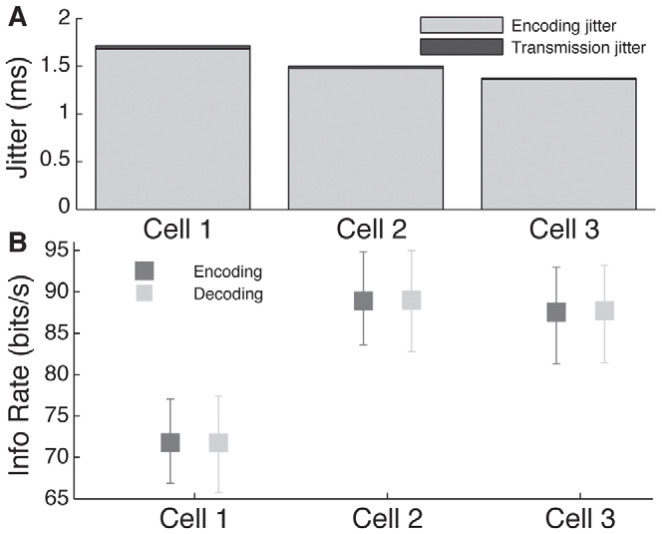
Measurement of jitter and information rates. *A*, Comparison of jitter at encoding end (light grey portion of bar) assessed over repeated presentations of stimulus, and transmission jitter (black portion of bar), measured in three different neurons. *B*, Mutual information rates for the three neurons in *A*, calculated at the encoding (light grey) and decoding (black) sites. Error bars represent Bayesian 95% confidence interval from CTW calculation.

To assess the significance of the above noise sources for a neuron's ability to transmit information to a postsynaptic target, we stimulated neurons with a repeating white noise air current and compared the magnitude of temporal jitter resulting from the transduction of the stimuli into spikes (encoding jitter) with temporal uncertainty that arises during conduction along the axon (transmission jitter). The results of this comparison for three neurons are shown in Figure 4A. The encoding jitter was assessed by determining the standard deviation of isolated single-spike responses across repeated stimulation [34]. Encoding jitter in these three cells ranged from 1.36 to 1.66 ms, while the transmission jitter ranged from 14.7 to 33.3 µs. This meant that the magnitude of the temporal spread due to conduction along the axon was 1.08% to 1.42% that of the stimulus-locked temporal uncertainty that occurred during spike generation.

The effects of the expansive non-linearity on short ISIs as well as the other noise sources we characterized could potentially have very large effects on the ability of these neurons to transmit information about stimuli to subsequent processing areas. To assess the cumulative deterioration of the signal during transmission from encoding site to decoding site, we estimated the mutual information rates of the same three neurons from Figure 4A. A significant decrease in information rates between the encoding and decoding sites would indicate that the various noise sources were degrading the ability of the neuron to signal changes in the stimulus to a postsynaptic cell. The results of our calculations are shown in Figure 4B. Note that the information measured at the encoding site includes the effects of the encoding jitter shown in Figure 4A, whereas the information measured at the decoding site includes both the encoding *and* transmission jitters, as well as all other noise sources which result from propagation along the axon. In all three neurons, changes in information rate between encoding and decoding site were smaller than the 95% CI of our estimate.

**Figure 5:**
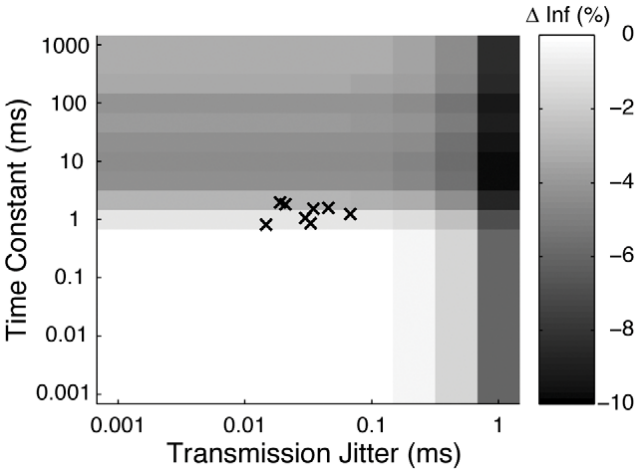
Model of change in information rates as a function of transmission jitter and expansive non-linearity. A surface depicting the percentage change in mutual information between encoding and decoding sites is shown in greyscale for model parametrized by the magnitude of the transmission jitter (Fig. 1C) and the time constant of the expansive nonlinearity (Fig. 2A). The parameter combinations measured in 8 neurons are shown with grey x's.

In order to further explore the ranges of conduction noise over which information transmission would be unaffected, we adopted our previous model [28] to include transmission jitter and the time constant for an expansive non-linearity (see Methods). For the sake of simplicity, we did not include effects due to a compressive non-linearity or conduction failure in our model, since these two effects were relatively small and only observable in some of our data. We tested the information rate for transmission jitters ranging from 10^−3^ to 10^1^ ms, and for time constants ranging from 10^−3^ to 10^3^ ms. The results of this calculation are shown in Figure 5. The grey scale represents a surface showing the change in information as a percentage of the information rate at the encoding site. The parameter combinations for the jitter and expansive non-linearity for each of the 8 neurons from which we recorded are shown as x's. Note that we clipped the x axis at transmission jitter = 1 ms in order to show greater contrast in the parameter regions neighboring the observed values. For the observed ranges of these noise parameters, our model predicts that there would be a modest 1%–3% loss in information, in agreement with the insignificant changes shown in Figure 4. Furthermore, our model predicts only small changes (∼7%) in information rates caused by the effects of the expansive non-linearity, even for time constants as long as 1 second. However, transmission jitter affects the information rate to a larger extent, decreasing transmission rates by 10% for 1 ms of transmission jitter (∼33% conduction error rate, following logic for Figure 1C).

### Changes in ISI Distributions Favor the Most Informative Intervals

**Figure 6:**
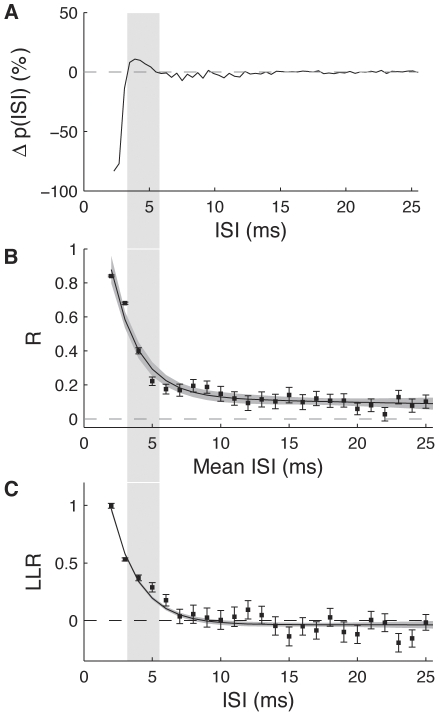
Change in ISI Distribution and Relation to Stimulus Coding. *A*, Percentage change in probability of ISI at decoding site relative to encoding site, same data as in figure 2D. The shaded region indicates ISIs that occur more frequently at the decoding site than at the encoding site. *B*, The correlation between first and second spikes of ISIs reliably elicited by repeated presentations of identical stimuli (“frozen noise”). *C*, The linearity of stimuli associated with doublet patterns of spikes with various ISIs, as assessed with log likelihood ratios. Data in *B* & *C* are from [28], reprinted with permission.

We have recently shown that pairs of spikes with small ISIs have greater precision and carry more information than spikes occurring in longer ISIs, and employ nonlinear strategies for encoding the stimulus [28]. We hypothesized that short ISIs in these cells could represent a separate information channel from single spikes, as happens in several bursting systems [35]–[37]. These short ISIs correspond to the regions most affected by the activity-dependent nonlinearities during transmission from encoding site to decoding site. The temporal relationship between the temporal precision of ISIs, linearity of coding associated with ISIs, and the changes of these ISIs during propagation are shown in Figure 6. 6A shows the percentage change in the ISI distribution between the encoding and decoding end, indicating that there is a relative increase in the number of ISIs between 3.6 and 5.2 ms. Panel B shows the correlation coefficient between neighboring ISIs over repeated presentations of an identical stimulus. For short ISIs (<10 ms) there is high correlation, indicating that these patterns are reliably produced in response to stimulation. Panel C shows the log likelihood ratio expressing how well a given stimulus can be predicted based on non-linear vs. linear models. This shows that the stimuli associated with short (<7 ms) ISIs are significantly non-linear. The interval over which ISI probability is increased at the decoding end correlates with the ISIs which have the highest temporal reliability and the most non-linear encoding.

## Discussion

To assess the significance of action potential conduction noise on neural coding, we have characterized several types of noise. This study represents the first effort to examine the effects of these noise sources using sensory stimulation, rather than current injection. These results demonstrate the constraints that several biophysical mechanisms impose on the capacity for neurons to transmit information to a postsynaptic target. We discuss these results in the context of previous measurements in other systems as well as potential impacts on mechanisms of coding and information transmission.

Our measurements of the transmission jitter were in general agreement with measurements from the sciatic nerve of frogs [9], as well as in proprioceptive afferents in crabs [38]. In contrast, the jitter we measured was much smaller than the ∼25% error rate over 2.7 mm of conduction measured in squid giant axons [12], but much higher than values predicted from models in that system, which were in the range of ∼0.02% error rate [15], [39]. In contrast, recent models of the very thin diameter axons in vertebrate cortex predict larger error rates (∼6%) than we see in the cercal system [19].

Temporal jitter can degrade the ability of nervous systems to transmit information about stimuli, causing optimal decoders to conflate the effects of temporal noise processes with variability in the driving stimulus [27]. To assess the coding significance of transmission jitter, we compared the variability in spike propagation times to the observed variability in spike initiation times. Our strategy was to assess the relative magnitude of transmission jitter with respect to the overall limiting temporal precision of the nerve cell: is the spike timing jitter insignificant with respect to the limiting temporal precision of the neuron as a whole, or is the magnitude of the jitter large enough to be a significant constraint on the cell's information encoding capacity?

Our raster-based measurement of encoding jitter yielded results of about 1.5 ms, in agreement with our previous characterization of these neurons [28]. The additional variability due to conduction along the axon was very much less than the temporal encoding precision, accounting for only ∼1% of total uncertainty at the decoding end. Therefore, the transmission jitter in these cells is not a significant determinant of their operation. It does not constrain the upper bound on the temporal precision with which patterns of action potentials could be decoded in this system, and hence would not constrain the limit of meaningful precision in the encoding operation. However, we note that this maintenance of information transmission rates during spike propagation need not be universal across all sensory systems. In the data shown here, the changes in transmission time are on the order of hundreds of microseconds. This may be typical for many neurons with axon diameter and length similar to the cricket, but signal degradation may be a much bigger problem for systems with longer-projecting axons. This is particularly important as changes in delay do not scale linearly with conduction distance [40].

Our observation of the dependence of conduction velocity on the time from the previous spike is in qualitative agreement with previous observations [7]–[9], [11]–[14], [16], [33], [41]. These effects arise from different aspects of the biophysics of ion channels and the natural fluctuations in ion concentration. For the very shortest interspike intervals (i.e. those that lie within the neuron's relative refractory period) the passage of a preceding spike leaves a large portion of the sodium channels in the inactive state. This leads to relatively longer time for the membrane potential to reach threshold, which in turn delays propagation along the axon. In contrast, for longer interspike intervals, the effect of a modest accumulation of potassium ions in the extracellular space leads to an increase in neuronal excitability, which leads to increased conduction velocity for action potentials [42]. These two phenomena have the effect of increasing the intervals of short ISIs while decreasing the length of moderate ISIs at the axon terminal. In the cricket system this leads to an increase in the probability of ISIs from 3.6 to 5.2 ms, at the cost of decreased probability for other intervals. However it is worth noting that in other systems such activity-dependent effects lead to much more drastic changes in ISI distributions between encoding and decoding end.

Through information-theoretic methods, we have demonstrated that the variability of axon propagation does not significantly limit the amount of information that can be decoded from these cells' neural activity. To address the possibility that the larger neuron population might have greater amounts of information loss during transmission, we developed a simple model to examine the relationship of the information transfer rate on the transmission jitter and the value of the activity-dependent time constant. Our model was in agreement with our data for compressive non-linearities and transmission jitter within the range we observed, and further predicted that wide ranges of the compressive nonlinearity would have little effect on information transmission. In contrast, it predicts that increases in the transmission jitter would have much larger effects – 10% of all information would be lost if transmission jitter was on the same order of magnitude as the encoding jitter (∼1 ms), and up to 70% of the information could be lost if transmission jitter was 10 ms.

The earliest efforts to quantify the effects of conduction noise on information transfer were primarily concerned with determining optimal ISI intervals for transmitting information [32], making them difficult to reconcile with our results. However, recent modeling work on propagation along tiny axons suggests a potentially much larger decrease in mutual information during conduction [19]. Since the magnitude of transmission jitter is relatively larger in thin axons, we would also predict a greater decrease in information transfer in such axons, and vice versa in larger axons. Indeed, in the relatively large axons of proprioceptive afferents in crabs, it has been shown that transmission jitter has relatively little effect on information transfer in comparison with encoding jitter [38]. This underscores the fact that the impact of noise on information transfer is likely to be neuron-specific, with large-diameter axons likely to be less affected by propagation noise.

A further mitigating factor that we didn't account for in our experiments is the known relationship between stimulus intensity and temporal precision of responses. It has been shown in previous experiments in the cricket cercal system that at very high stimulus intensities the neurons become temporally locked to specific stimulus events [43]. It is possible that at such high stimulus intensities (and consequent low temporal variability in spike generation), the contribution of propagation noise to total variability of spike timing could become larger relative to the values reported here. Conversely, at very low stimulus intensities, noise sources extrinsic to the stimulus can actually improve the encoding capacities of neurons through the phenomenon of stochastic resonance [44]. An interesting line of further research would be to examine the relationship between stimulus intensity and the contribution of variability in spike propagation to the information content about the stimulus that can be extracted from the spike train.

In total these observations reveal a potential trade-off between neurons that employ temporal vs. rate coding strategies. Temporal coders, such as the neurons studied here, must limit the extent to which conduction variability and non-linearities act to change the temporal patterning of spiking activity between encoding and decoding regions. Our results show multiple ways that this can be accomplished. First, the use of axons with large diameters reduces the effect of that the stochastic nature of single ion channels has on spike conduction. Second, since conduction noise compounds over the length of the axon, it would be important to keep the transduction distance to a relative minimum. Finally, it is important to minimize the non-linear effects of the supernormal and subnormal periods, which can be done through the selection and distribution density of ion channels in the axon [40]. In contrast, neurons using rate coding are free to instead exploit these various non-linearities to implement coding strategies such as contrast enhancement [45].

It will be interesting to see to what extent the cricket cercal system uses this trade-off in coding strategies. The neurons in the full system contain ∼20 bilateral pairs of axons of various diameter [20], [21], [23], [46], [47], from which recordings can be made both near their spike initiation zone as well as their axon terminals several millimeters away. In addition, the neurons in the system exhibit varying degrees of temporal precision [48], personal observation), which would provide a convenient platform for examining this question. Further, these new results contain intriguing implications for the use of temporal coding in this system [28]. The end result of the conduction non-linearities is to produce a resonant peak at ISIs<6 ms, which could lead to improved transmissibility of information to the next layer of the system through spike-timing dependent mechanisms [49]–[51] and the appropriate tuning of synapses [52]. In fact, there is evidence from cockroaches that short ISI bursts in this system preferentially lead to spiking in a postsynaptic motor neuron, whereas other patterns of spikes do not [53]. A fruitful line of future research will be to determine the ranges over which patterns of spikes in giant interneurons of the cercal system lead to spiking in their postsynaptic targets, and particularly whether such spike patterns result from behaviorally relevant stimuli.
